# SNP Discovery in the Transcriptome of White Pacific Shrimp *Litopenaeus vannamei* by Next Generation Sequencing

**DOI:** 10.1371/journal.pone.0087218

**Published:** 2014-01-30

**Authors:** Yang Yu, Jiankai Wei, Xiaojun Zhang, Jingwen Liu, Chengzhang Liu, Fuhua Li, Jianhai Xiang

**Affiliations:** 1 Key Laboratory of Experimental Marine Biology, Institute of Oceanology, Chinese Academy of Sciences, Qingdao, China; 2 University of Chinese Academy of Sciences, Beijing, China; Chinese Academy of Fishery Sciences, China

## Abstract

The application of next generation sequencing technology has greatly facilitated high throughput single nucleotide polymorphism (SNP) discovery and genotyping in genetic research. In the present study, SNPs were discovered based on two transcriptomes of *Litopenaeus vannamei* (*L. vannamei*) generated from Illumina sequencing platform HiSeq 2000. One transcriptome of *L. vannamei* was obtained through sequencing on the RNA from larvae at mysis stage and its reference sequence was *de novo* assembled. The data from another transcriptome were downloaded from NCBI and the reads of the two transcriptomes were mapped separately to the assembled reference by BWA. SNP calling was performed using SAMtools. A total of 58,717 and 36,277 SNPs with high quality were predicted from the two transcriptomes, respectively. SNP calling was also performed using the reads of two transcriptomes together, and a total of 96,040 SNPs with high quality were predicted. Among these 96,040 SNPs, 5,242 and 29,129 were predicted as non-synonymous and synonymous SNPs respectively. Characterization analysis of the predicted SNPs in *L. vannamei* showed that the estimated SNP frequency was 0.21% (one SNP per 476 bp) and the estimated ratio for transition to transversion was 2.0. Fifty SNPs were randomly selected for validation by Sanger sequencing after PCR amplification and 76% of SNPs were confirmed, which indicated that the SNPs predicted in this study were reliable. These SNPs will be very useful for genetic study in *L. vannamei*, especially for the high density linkage map construction and genome-wide association studies.

## Introduction

*Litopenaeus vannamei* (*L. vannamei*) is widely cultured in Asia, South and North America. According to the statistics on the global aquaculture production by FAO in 2011, the whole production of *L. vannamei* occupied 76% of the world penaeid shrimp production. In order to understand the molecular mechanism for desired traits, molecular genetics research such as linkage map construction [1]–[4], quantitative trait loci (QTL) analysis, trait-genotype association study [5] have been conducted to allow marker-assisted selection. Although *L. vannamei* is an important and worldwide shrimp species in aquaculture, available genetic markers in public database for this species was limited.

Single nucleotide polymorphisms (SNPs) are the most abundant type of DNA sequence polymorphism which has been proved to be useful in genetic studies [6]. It has been applied in quantitative trait loci (QTL) mapping and genome wide association studies (GWAS) in model organisms and human [7]–[10]. In aquaculture species, SNP markers are becoming more important for linkage map construction and association studies [11]–[14]. In recent years, more efforts have been made for SNP discovery in *L. vannamei* [15]–[17]. However, these SNPs were still insufficient for high density SNP chip construction and genome wide association studies.

Next generation sequencing technologies have made high throughput SNP discovery feasible for non-model species [18]–[20]. Recently, transcriptome sequencing has become the major method for SNP discovery [18]. Through transcriptome sequencing, functional genes could be sequenced at high coverage, which ensured full-scale SNP discovery in coding genes with high accuracy. Large amount of SNPs have been developed by next generation transcriptome sequencing in aquaculture species such as catfish [21], Atlantic Cod [11], oyster [22], half-smooth tongue sole [23], Atlantic Herriing [18], silver carp [24], common carp [25] and Altantic Salmon [26]. In *L. vannamei*, transcriptome sequencing was applied in shrimp larvae, TSV-infected and non-infected individuals using next-generation sequencing technique [27], [28]. It supplied a large amount of gene information related to development and disease resistance. However, SNPs discovery in transcriptome data by next generation sequencing was not reported in *L. vannamei* till present.

In the present study, SNPs were predicted by analysis of reads from two transcriptomes of *L. vannamei* and the characterization of these SNPs was analyzed. The predicted SNPs in this study will be very helpful for further genetic studies in *L. vannamei*, especially for the high density linkage map construction and genome wide association studies.

## Materials and Methods

### Transcriptome Sequencing and Reads Collection

Hundreds of *L. vannamei* larvae at developmental stage of mysis were collected from Guangtai Marine breeding company in Hainan province, China. They were frozen in liquid nitrogen immediately and stored in −80°C before RNA isolation. Total RNA was isolated using RNAiso Plus (Takara, Japan) following manufacturer’s protocol. The RNA amounts and quality was estimated using NanoDrop 1000 spectrophotometer (Nano-Drop Technologies, USA). Sequencing of RNA extracted from mysis larvae was conducted in BGI (Shenzhen, China) using the paired-end RNA-Seq method [29]. The detail procedure was the same as described previously [30]. The reads obtained through this way were defined as M transcriptome in the present study.

The reads of 100 larvae of *L. vannamei* at 20 days post spawning, which was deposited in NCBI with the Sequence Read Archive (SRA) accession number of SRR346404 [28], were downloaded and defined as P transcriptome. All reads were filtered with NGS QC Toolkit using default settings before further analysis.

### SNP Detection

The reads from M transcriptome were *de novo* assembled using Trinity [31] and the assembled sequence was used as reference in this study. Firstly, SNPs were detected using reads of M transcriptome and P transcriptome separately. Short reads of M and P transcriptome were separately mapped to the reference using BWA version 0.5.9 (http://bio-bwa.sourceforge.net/) with the default settings except for no gap tolerance. The software package SAMtools (http://samtools.sourceforge.net/) was used to convert sequence alignment/map (SAM) file to sorted binary alignment/map (BAM) file [32]. The command Rmdup was used to remove duplicates and SNPs were detected using mpileup in SAMtools using the following parameter: −6 (Illumina 1.3+ encoded quality score) -g -u (Compute genotype likelihoods and generate binary call format) -C 50–D (Output per-sample read depth). SNPs were called by Bcftools. SNPs with quality score more than 20 and read depth over 10 were filtered as high quality SNPs. Since prediction accuracy of SNPs is dependent on sequence coverage [33], we combined the reads of the two transcriptomes (M+P transcriptome) in order to improve sequence coverage. SNPs detection was conducted again using the combined reads with the same method. The further characteristic analysis was based on the SNPs predicted in the reads of M+P transcriptome.

### Statistics of SNP Information

Mapped reads ratio (MRR) to the reference in each dataset was calculated by applying flagstat command of SAMtools software to the BAM file. SNP frequency was calculated by dividing the total length of reference by total number of SNPs. Transition verse transversion ratio was calculated by analyzing each type of DNA substitution. SNP depth (DP) and Minor Allele Frequency were also extract from the result file of SAMtools. SNP classification was obtained by a Perl script used in previous study [17].

### Functional Annotation of SNPs

The unigenes containing SNPs were annotated using Basic Local Alignment Search Tool (BLAST) against to NCBI non-redundant (nr) database by BLASTX(E-value cut off <1.0e−5). The BLAST results were utilized by Blast2GO to annotate the unigenes with GO terms of biological processes, molecular functions, and cellular components [34]. Annotated information was imported into BGI WEGO program (http://wego.genomics.org.cn) in WEGO native format to plotting GO annotation results. KEGG pathways were assigned to unigenes containing SNPs using the online KEGG Automatic Annotation Server (KAAS) (http://www.genome.jp/tools/kaas/) [35]. KEGG Orthology (KO) assignment was applied using Bi-directional Best Hit (BBH) method.

### SNP Validation

In order to validate the accuracy of SNPs prediction, 50 SNPs were randomly selected for SNP validation using DNA as templates. Primers were designed to amplify the flanking sequence of selected SNPs using Primer 3 with fragment length of 200 bp. Primers were synthesized in Sangon Biotech (Shanghai, China). Eight DNA pools made by 96 individuals were used as templates for amplification. The amplified PCR products were sequenced by ABI Prism 3730 sequencer (Applied Biosystem, USA) and sequencing results were analyzed by BioEdit version 7.0.5.3 (http://www.mbio.ncsu.edu/BioEdit/bioedit.html).

## Results

### Generation of Expressed Short Reads

After filter using NGS QC Toolkit, a total of 53,902,786 high quality reads with 90 bp were generated from M transcriptome (NCBI Sequence Read Archive accession number SRR1039534). A total of 24,680,276 high quality reads with 90 bp were obtained from P transcriptome (Table 1).

**Table 1:** Statistics of raw reads with high quality and mapped reads ratio of M transcriptome and P transcriptome.

Dataset	Mapped reads	Raw reads	Mapped reads ratio
**M^a^**	46,803,406	53,902,786	86.83%
**P^b^**	17,694,976	24,680,276	71.70%

M^a^ Reads from M transcriptome.

P^b^ Reads from P transcriptome.

### Reads Mapping and SNP Detection

**Figure 1:**
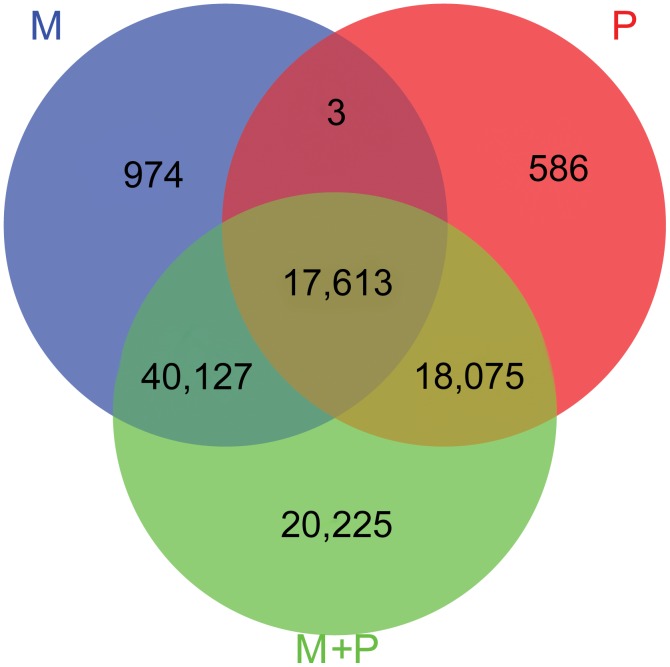
Venn diagram of SNPs discovered using reads of the three datasets. M represented SNPs discovered using reads from transcriptome of L.vannmei at developmental stage of mysis, P represented SNPs discovered using reads from transcriptome of L.vannmei at developmental stage of post larva. M+P represented SNPs discovered using the reads of the two transcriptomes together.

*De novo* assembly of the independent short reads from M transcriptome generates 66,215 unigenes with an average length of 705 bp. A total of 86.83% and 71.70% of the short reads from M and P transcriptome were mapped to the reference separately (Table 1). The alignment file was used for SNP detection using SAMtools [32]. In order to get more reliable SNPs, those with quality score over 20 and the reads depth over 10 were regarded as high quality SNPs. A total of 58,717 and 36,277 putative SNPs with high quality were predicted in the M transcriptome and P transcriptome, respectively. Using the two sets of data together (M+P transcriptome), a total of 96,040 SNPs with high quality were predicted. Comparison of SNPs predicted in M transcriptome, P transcriptome and M+P transcriptomes were shown in Figure 1. It showed that 49% of the SNPs in P transcriptome were found to be the same as that in P transcriptome, 98% of the SNPs predicted in M transcriptome or P transcriptome could be found in the predicted SNPs in M+P transcriptomes. In the M+P transcriptome, other 20,225 SNPs were predicted besides the common SNPs to those in M transcriptome or P transcriptome. We analyzed the heterozygosity and homozygosity of those 20,225 SNPs in the two transcritpomes. It showed that most of the SNPs were heterozygous in each transcriptome. As the SNPs discovered by combined data supplied more information, further analysis was conducted on the SNPs predicted in the M+P transcriptomes.

The estimated SNP frequency was 0.21% (one per 476 bp). Within the identified SNPs, more transitions substitution (66.8%) were found than transversion substitution (33.2%) (Table 2). In terms of transition substitution, the amount of A/G transitions was similar to that of C/T transition. In terms of transversion substitution, the frequency of four types (G/C, G/T, A/C and A/T) was equal. The estimated ratio for transition to transversion was 2.0.

**Table 2:** Statistics of transition and transversion type in the total SNPs.

Type	Transition		Transversion			
	GA	CT	GC	GT	AC	AT
Number	32,209	32,695	6,404	7,191	7,388	10,153
Percentage	33.5%	33.3%	6.9%	7.9%	7.7%	10.8%

### Read Depth and Minor Allele Frequency Distribution

**Figure 2:**
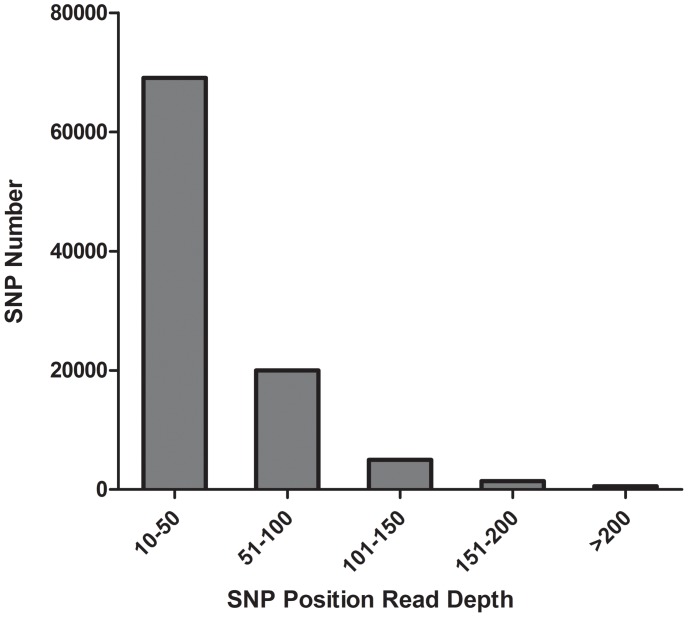
Statistics of read depth in each SNP in *L. vannamei*. The horizontal axis represented the read depth of SNPs, the vertical axis represented the number of SNPs with the corresponding read depth. The average read depth was 44.

As the read depth in SNPs position was closely related to the prediction accuracy of SNPs [30], the statistics of read depth for each SNP was calculated and plotted (Figure 2). It showed that the estimated average read depth was 44. SNPs with read depth between 10 and 50 account for the majority (72%) while SNPs with read depth range from 51–100 account for nearly 20%.

**Figure 3:**
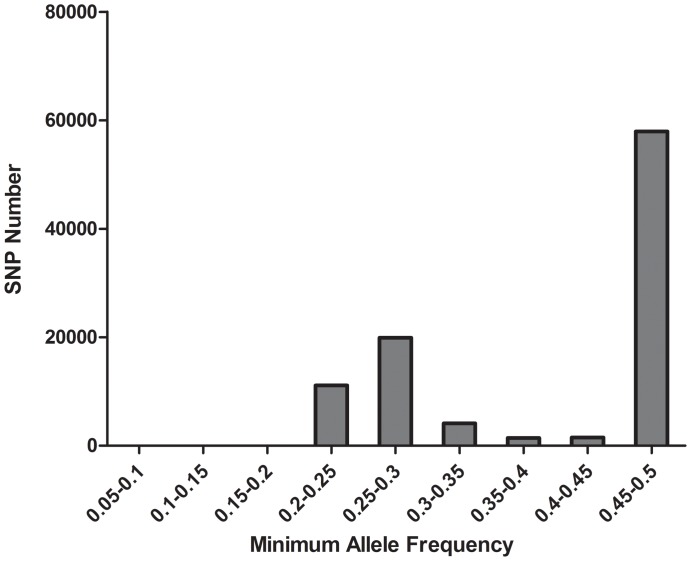
Statistics of minor allele frequency of total discovered SNPs in *L. vannamei*. SNP number with MAF below 0.2 was too small to be observed in the column diagram.

Minor allele frequency (MAF) was calculated based on the sequence data. As only SNPs with MAF more than 0.05 are regarded as true SNPs, those with more than 0.05 MAF were analyzed. SNPs with MAF range from 0.45–0.5 accounts for 60% of the total SNPs (Figure 3).

### SNP Classification

As shown in Table 3, totally 57,793 SNPs were annotated. Among these, 34,371 SNPs were located in the open reading frame including 5,242 non-synonymous SNPs and 29,129 synonymous SNPs, 23,422 SNPs were located in 5′ or 3′ UTR region. As there were limited data of related species, 38,247 SNPs were not annotated.

**Table 3:** Classification of identified SNPs using a manual Perl script.

SNP classification	SNP number
Non-synonymous SNP	5,242
Synonymous SNP	29,129
5′ or 3′ UTR	23,422
Not annotated	38,247
Total	96,040

### Assessment of SNP Distribution

**Figure 4:**
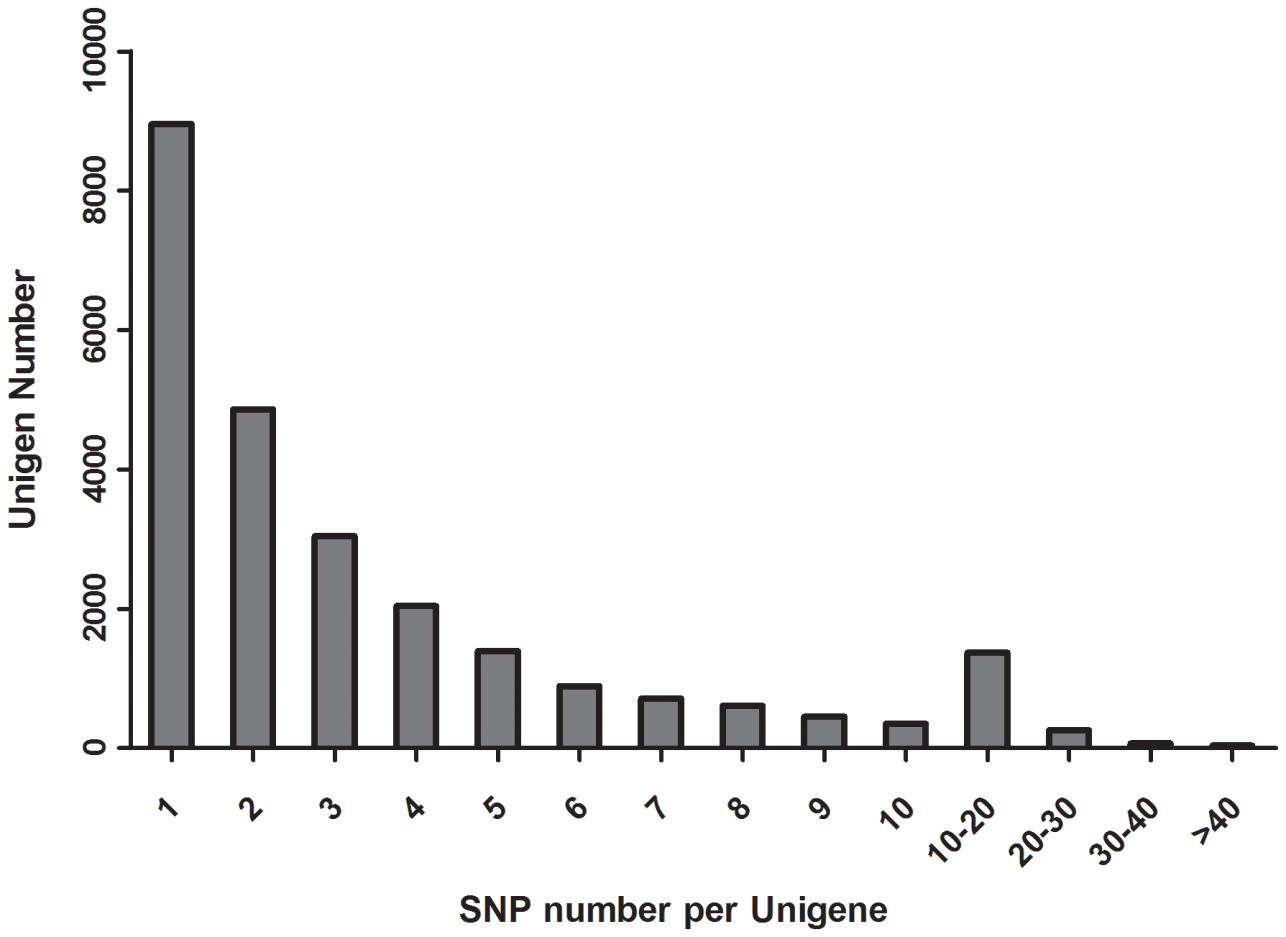
The distribution of SNPs in unigenes. The horizontal axis represented SNP numbers per unigene. The vertical axis represented the number of unigenes.

**Figure 5:**
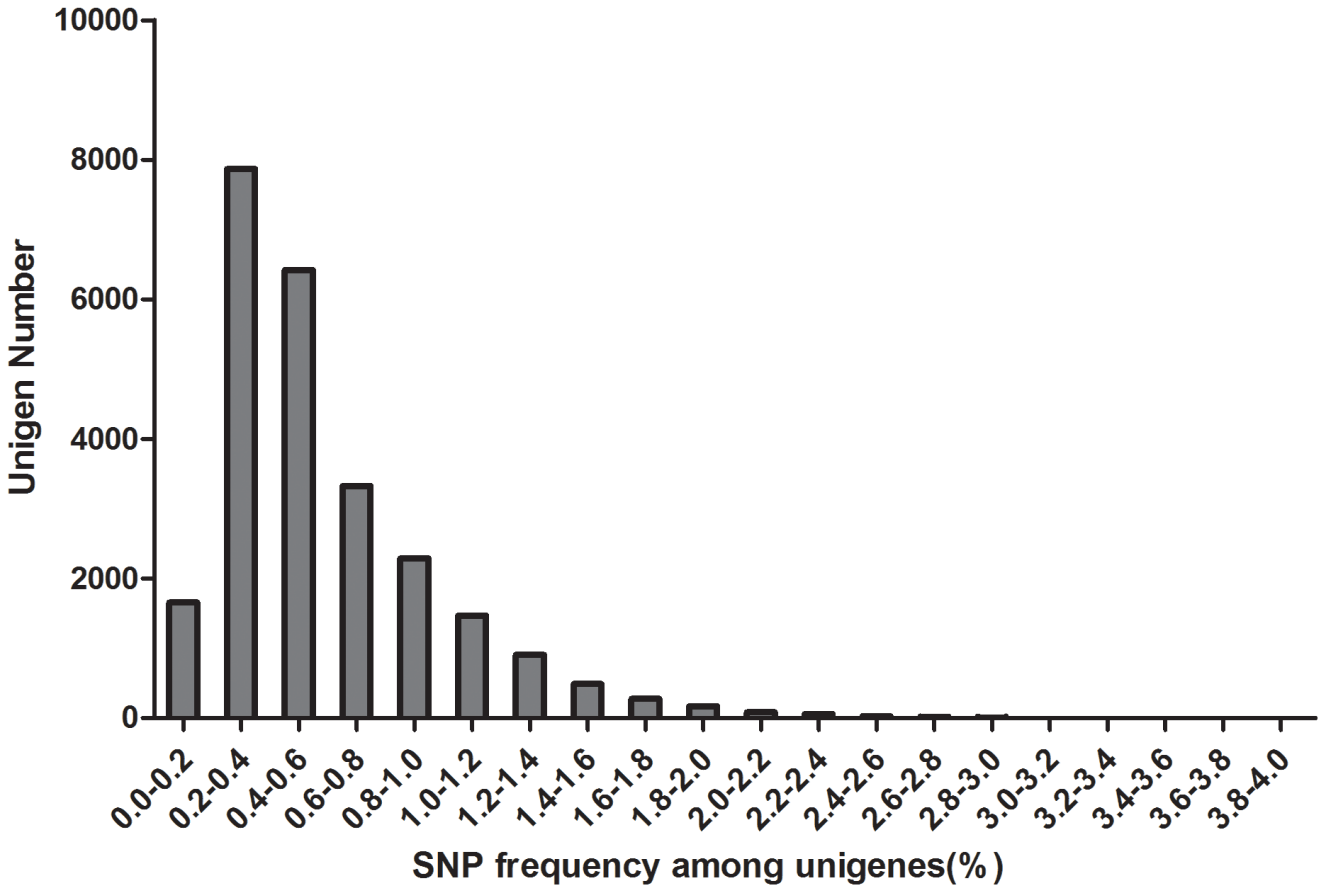
The frequencies of SNPs in unigenes. The frequencies of SNPs in each unigene was calculated by dividing unigene length by SNPs number per unigene. Number of unigenes with SNP frequency over 0.030 was too small to be observed in the column diagram.

SNP distribution among unigenes is important when considering the marker density and genome coverage using SNP marker [21], especially when these SNPs were used for linkage map construction. In this study, we found that all the SNPs were distributed in 25,071 unigenes (38% of the total unigenes). Among these 25,071 unigenes, unigenes with 1 SNP were more common and those with no more than 10 SNPs occupied 93% of total unigenes. A total of 1,740 unigenes containing more than 10 SNPs were observed. The detailed SNPs distribution among those unigenes was shown in Figure 4. In order to investigate the mutation rate among unigenes, the SNP frequency within unigenes was calculated and shown in Figure 5. The top twenty annotated unigenes with the highest SNP frequency were listed in Table 4.

**Table 4:** Annotation of the top twenty unigenes with the highest SNP frequency.

Unigenes	SNP frequency	Nr Annotation
Unigene15570	0.02253	viral A-type inclusion protein [*Trichomonas vaginalis G3*]
Unigene14752	0.022634	PREDICTED: protein lethal(2)essential for life-like [*Acyrthosiphon pisum*]
Unigene1532	0.02268	early cuticle protein 5 [*Callinectes sapidus*]
Unigene3466	0.023529	hypothetical protein [*Monosiga brevicollis MX1*]
Unigene2230	0.023564	hypothetical protein DDB_G0288123 [*Dictyostelium discoideum AX4*]
Unigene2037	0.024038	anti-lipopolysaccharide factor [*Macrobrachium rosenbergii*]
Unigene17231	0.024096	hypothetical protein [*Monosiga brevicollis MX1*]
Unigene27996	0.02446	dimethylglycine dehydrogenase, isoform CRA_a [*Homo sapiens*]
Unigene6364	0.024476	GD25285 [*Drosophila simulans*]
Unigene18738	0.024725	hypothetical protein [*Monosiga brevicollis MX1*]
CL1560.Contig3	0.026201	Spz3 [*Litopenaeus vannamei*]
Unigene7779	0.026201	GM24425 [*Drosophila sechellia*]
Unigene14736	0.026415	PREDICTED: uncharacterized protein C15orf39-like isoform 1 [*Pongo abelii*]
CL1752.Contig1	0.026477	PREDICTED: hypothetical protein [*Strongylocentrotus purpuratus*]
Unigene27125	0.02649	hypothetical protein, conserved in P. knowlesi [*Plasmodium knowlesi strain H*]
Unigene6916	0.028302	Hypothetical protein CBG18078 [*Caenorhabditis briggsae*]
CL3267.Contig1	0.028871	hypothetical protein TcasGA2_TC002455 [*Tribolium castaneum*]
Unigene7603	0.030303	hypothetical protein BRAFLDRAFT_187367 [*Branchiostoma floridae*]
CL3620.Contig1	0.03125	crustacyanin-A, partial [*Cherax quadricarinatus*]
Unigene893	0.034392	heat shock protein 21 [*Macrobrachium rosenbergii*]

### SNP Annotation and Functional Analysis

**Figure 6:**
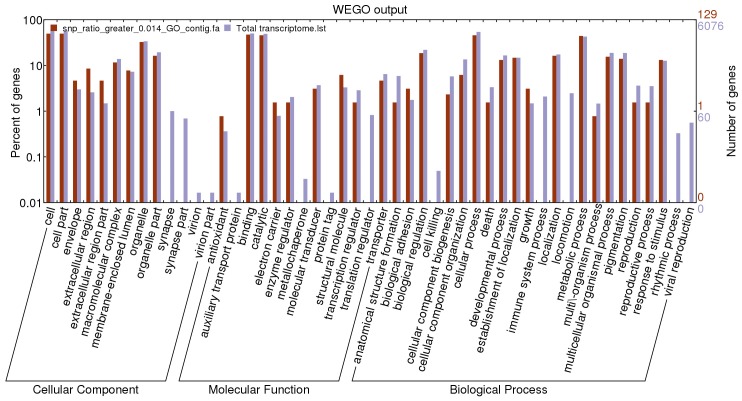
Gene ontology of all the annotated unigenes and unigenes with SNP frequency more than 0.014. The blue column represented gene ontology of all unigenes containing SNPs. The red column represented gene ontology of unigenes with SNP frequency more than 0.014.

Among the 25,071 unigenes containing SNPs, 13,591 unigenes (54%) had significant hit to the protein in the non-redundant (nr) database. The unigenes were annotated by the corresponding top best BLASTx hit. After the Gene Ontology annotation, 12,978 unigenes (52%) were assigned with one or more GO ID. The plotted GO annotations of these annotated unigenes were shown in Figure 6.

The unigenes with higher SNP frequency (more than 0.014) were separately extracted from the annotated file and their GO annotation were plotted together with those of total SNPs (Figure 6). Genes in synapse, synapse part, virion and virion part in cellular component category tended to be less polymorphic. Genes in auxiliary transport protein, metallochaperone, protein tag and translation regulator in molecular function category tended to be less polymorphic. Genes in cell killing, immune system process, locomotion, rhythmic process and viral reproduction process in biological process category tended to be less polymorphic.

**Figure 7:**
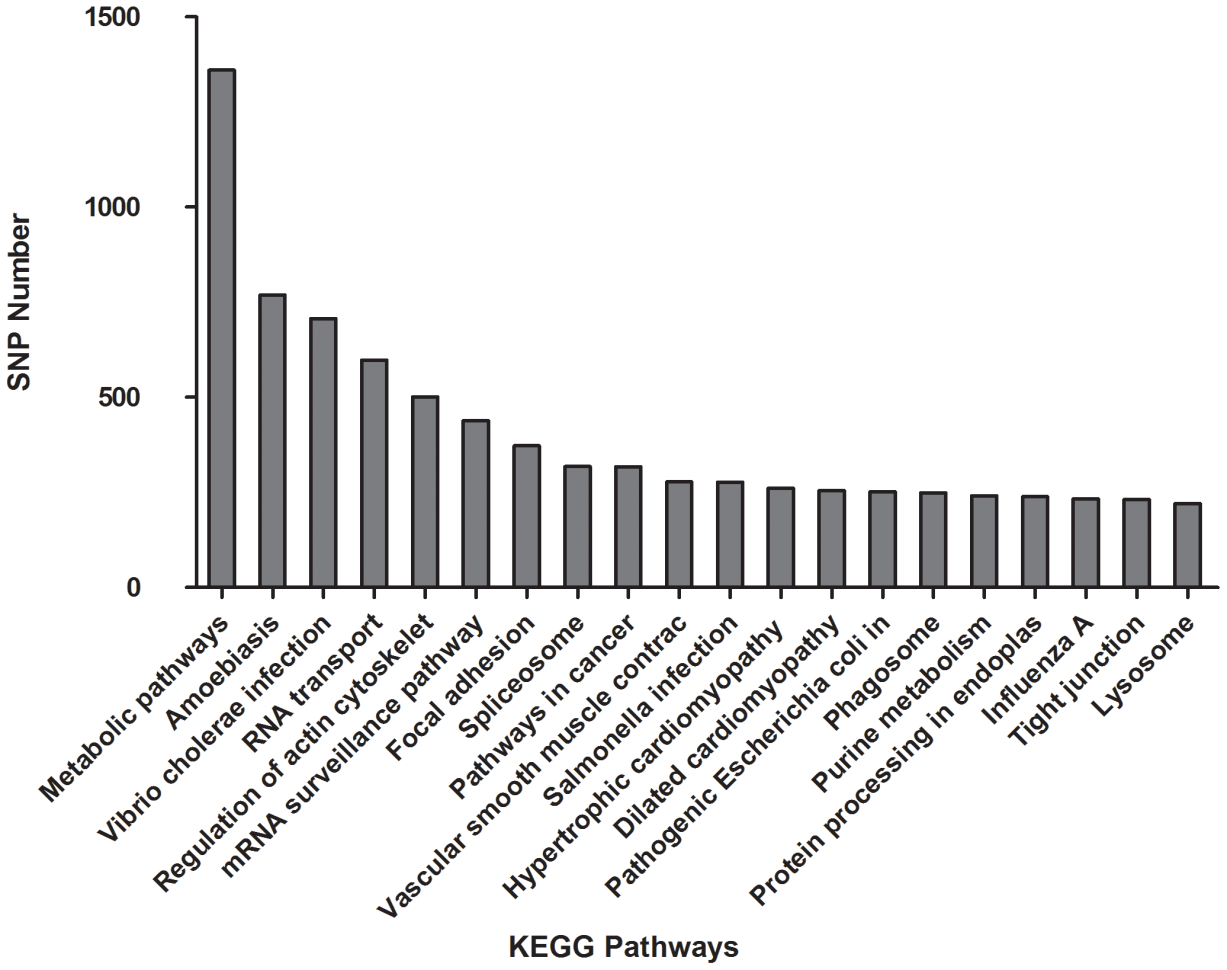
The top 20 KEGG pathway classification of assigned SNPs. The horizontal axis represented KEGG pathway annotation. The vertical axis represented the number of SNPs assigned to the corresponding KEGG pathway.

KEGG analysis showed that 10,803 (43% of total) unigens containing 23,553 SNPs could be annotated by KEGG database. These unigenes could be assigned to 254 KEGG pathways (Table S1). The top 20 assignment KEGG pathways were shown in Figure 7.

### SNP Validation

Among the 50 primer pairs designed for SNP validation, 34 could amplify target sequences. Within these amplified sequences, 26 SNPs were validated (Table S2). The estimated predicting accuracy reached 76%. For SNPs with quality score over 90, the predicting accuracy could be up to 87.5%.

## Discussion

The purpose of the present study is to develop a large amount of convinced and gene specific SNPs for *L. vannamei* through transcriptome sequencing. As next generation sequencing could enable the deep and efficient probing of transcriptome [36], most functional genes at the corresponding developmental stage could be involved in the transcriptome. It ensures a sufficient resource for gene-associated SNPs discovery. To generate more SNP information of *L. vannamei*, two sets of transcriptome data were used in this study. A total of 58,717 and 36,277 SNPs with high quality were predicted by using these two sets of data separately. Using the reads of two transcriptomes together, we detected 96,040 SNPs including 20,225 SNPs which was not detected by using the two transcriptomes separately. Heterozygosity and homozygosity analysis of these 20,225 SNPs indicate most of these SNPs were polymorphic in each transcriptome.

The overlap analysis of SNPs discovered by two transcriptomes separately showed that only half of the SNPs were identical. The identical SNPs may refer to SNPs which could be easily transferred in this species. This result indicated that *L. vannamei* may endow a moderately high genetic diversity. Similar reports were published in *Streblospio benedicti* where population differentiation were observed between different populations [37].

MAF is a relative estimation of true allele frequency in the population [38]. The distribution of minor allele frequencies in this study deviated obviously from a uniform distribution, with an excess of alleles at high frequency. SNPs with MAF range from 0.45 to 0.5 accounts for 60% of the total SNPs, which is different from the data reported previously [39]. It inferred that the individuals used for transcriptome sequencing contained a high number of heterozygous loci. It is consistent with the results discovered by genome sequencing [40]. The estimated SNP frequency in *L. vannamei* is one per 476 bp, which is lower than European hake (1/137 bp) [22], Eastern oyster (1/60 bp) [41] and higher than Atlantic cod (1/516 bp) [11], Atlantic salmon (1/614 bp) and Salt Marsh Beetle (1/898 bp) [42]. The SNP frequency was moderately higher in the reported species. The transition/transversion ratio (2.0) was also higher than Pacific oyster (1.3) [43] and Drosophila(1.5) [44].

Read depth is a key parameter affecting the predicting accuracy of SNPs [30]. One advantage of Illumina sequencing platform is the higher read depth comparing to 454 sequencing platform. It ensures the detection of true SNPs. In this study, average read depth of SNP position was 44 which was enough to guarantee the accuracy of discovered SNPs. It also could ensure that most of expected SNPs in the sequenced population could be detected [33]. SNPs with much higher read depth should be excluded since too high read depth might be caused by paralogous sequence variants [18].

Another advantage of SNP discovery using transcriptome data is to find the SNPs directly associated with interested traits, such as disease resistance or growth advantages. Researchers primarily focused on non-synonymous coding SNPs (nsSNPs) as those SNPs might influence the protein activity directly. Reports on human genome wide association studies (GWAS) showed that the synonymous SNPs might play the same role as those nsSNPs [45]. SNPs in 3′ or 5′ un-translated regions were also very important since some of them might lead to changes in mRNA binding sites [46], [47]. Most of the annotated SNPs predicted in the present study were located in UTR region, and only 6,869 SNPs were non-synonymous SNPs. These SNPs are possibly to be used in the further genome wide association studies and genome selection breeding program of *L. vannamei*.

The SNP frequency in each unigene was calculated in this study. The SNP frequencies ranged from one per 1.4 kbp to one per 26 bp. The high polymorphic unigenes detected in this study could be used in the population diversity or population differentiation analysis. We arbitrarily categorized unigenes with SNP frequency over 0.014 as higher polymorphic genes. Gene Ontology analysis showed that some components tend to be less polymorphic.

Among 50 primers used for SNP validation, 34 primer pairs could amplified target sequence. As we used genome DNA for validation, primers may locate in boundary of exon and intron which resulted in failed amplification. Another reason for failed amplification may be large size intron insert between primer pair. We also found that the quality score of SNPs influenced the validation result. The accuracy for SNPs with quality score over 90 was higher than that with quality score over 20. It was reported previously that when selecting SNPs in genetic studies, we should consider the allele frequency of the SNPs [21]. Considering these together, both MAF and quality score should be in consideration in further genetic research.

## Conclusion

In this study, next generation sequencing reads from two transcriptomes of *L. vannmei* were used for SNP discovery. A total of 96,040 high quality SNPs were predicted using the reads of the two transcriptomes together. Within those SNPs, approximately half could be annotated. The read depth and MAF analysis showed these predicted SNPs were accurate and common in this species. Besides, a moderately high genetic diversity and high heterozygosity in *L. vannmei* were found through characteristic analysis of SNPs. Overall, the SNPs predicted in this study will be useful in the genome wide association studies and whole genome selection studies.

## Supporting Information

Table S1 **KEGG pathway of annotated SNPs.**
(XLSX)

Table S2 **The SNPs validated by Sanger sequencing.**
(XLSX)
